# Potential risk of neoplasms with fezolinetant: clinical evidence, mechanisms, and post-marketing implications

**DOI:** 10.1210/jendso/bvag082

**Published:** 2026-04-01

**Authors:** Emma Boretti, Jean-Michel Dogné, Jonathan Douxfils

**Affiliations:** Clinical Pharmacology and Toxicology Research Unit, Namur Research Institute for Life Sciences (NARILIS), Faculty of Medicine, University of Namur, Namur 5000, Belgium; Clinical Pharmacology and Toxicology Research Unit, Namur Research Institute for Life Sciences (NARILIS), Faculty of Medicine, University of Namur, Namur 5000, Belgium; Clinical Pharmacology and Toxicology Research Unit, Namur Research Institute for Life Sciences (NARILIS), Faculty of Medicine, University of Namur, Namur 5000, Belgium; Department of Biological Hematology, CHU Clermont-Ferrand, Hôpital Estaing, Clermont-Ferrand 63100, France

**Keywords:** fezolinetant, menopause, neoplasms, neurokinin-3, hot flashes

## Abstract

In 2023, the Food and Drug Administration (FDA) and the European Medicines Agency (EMA) approved fezolinetant (Veozah^®^ in the United States, Veoza^®^ in Europe), a novel neurokinin 3 receptor (NK3R) antagonist and first-in-class nonhormonal drug for treating moderate to severe menopausal vasomotor symptoms (VMS). While its efficacy and safety have been demonstrated through the SKYLIGHT and MOONLIGHT programs, concerns were highlighted regarding a potential increased risk of neoplasms. Although regulatory agencies initially dismissed that risk, emerging data from meta-analysis and pooled analysis consistently showed a significant increase in the risk of nonbenign neoplasms. These findings raise important questions regarding a potential causal link between NK3R antagonists and cancer. By blocking NK3R, fezolinetant can reduce kisspeptin secretion, a ubiquitous peptide known for its antimetastasis function. Furthermore, NK3R blockade may induce compensatory activation of the neurokinin 1 receptor (NK1R), which has been implicated in tumor proliferation, angiogenesis, and metastasis. Because of these mechanistic concerns, long-term safety studies and investigations on the NK3R pathway are essential to clarify the neoplastic risk profile of fezolinetant. This review is based on a PubMed/MEDLINE search using specific keywords, complemented by screening of regulatory documents and citation tracking.

Fezolinetant is a first-in-class neurokinin 3 receptor (NK3R) antagonist recently approved for the treatment of moderate to severe vasomotor symptoms (VMS) associated with menopause. While its clinical efficacy has been demonstrated in large-scale phase-3 trials, an unexpected numerical imbalance in the incidence of neoplasms has emerged across both United States and European regulatory evaluations. Despite these signals, neither the Food and Drug Administration (FDA) nor the European Medicines Agency (EMA) has required specific postauthorization safety (PASS) studies or risk minimization strategies to address this risk. This is concerning, given its novel mechanism and lack of long-term safety data for NK3R antagonists. Additionally, emerging evidence from pharmacological studies suggests plausible indirect mechanisms through which NK3R blockade could interfere with tumor-suppressive pathways, including kisspeptin signaling and immune surveillance.

This manuscript reviews clinical, regulatory, and pharmacological data surrounding fezolinetant safety profile, explores the biological plausibility of a neoplastic risk, and proposes recommendations to enhance its safe use in clinical practice based on a specific PubMed/MEDLINE search using specific keywords, complemented by screening of regulatory documents and citation tracking. Both preclinical and clinical studies were reviewed to assess mechanistic plausibility and evaluate the consistency of reported safety signals.

## Regulatory review

### Overview

On May 12, 2023, the FDA approved fezolinetant (Veozah^®^), a NK3R antagonist in kisspeptin, neurokinin, dynorphin (KNDy) neurons, for the treatment of moderate to severe VMS due to menopause [1]. The EMA also granted a positive opinion on fezolinetant (Veoza®) on October 13, 2023, based on the results of the SKYLIGHT program, which evaluated its efficacy and safety at doses of 30 and 45 mg [2-5].

Both agencies noted an imbalance in neoplasm cases during their evaluations [5, 6]. The FDA-Clinical Review (review completion date, November 23, 2022) mentioned a dose-dependent numeric imbalance in the incidence of malignancy with 20 cases over 2203 fezolinetant-treated participants vs 2 cases over 952 placebo participants [6]. Among these 22 cases, 18 were further discussed and presented in a summary table. Based on the apparent imbalance in the incidence of malignancy in treatment compared to placebo groups, the FDA review team consulted the Division of Oncology III. The response from the Division Oncology III is added to the FDA-Clinical Review as a memorandum dated January 23, 2023. The Division Oncology III was not able to position itself on the risk [6]. They suggest an additional review from the pharmacology toxicology team, highlighting NK3R involvement in oral squamous cell carcinoma (OSCC) in which SB222200, a selective NK3R antagonist, significantly suppressed the radiographic osteolytic lesion and tumorigenesis [7]. The FDA Nonclinical review document provides a review addendum dated April 20, 2023, in which the reviewers acknowledged they did not identify a possible mechanism for an increased risk of neoplasm with fezolinetant. They also checked whether fezolinetant met any of the criteria of the key characteristics of carcinogens as presented in the International Agency for Research on Cancer (IARC) monograph [8]. The FDA mentions that the 2-year carcinogenicity study did not reveal significant findings. However, there was a statistically significant dose–response relationship in tumor incidences for benign follicular cell adenomas in the thyroid with increased fezolinetant dose across the vehicle control and the treated groups of female rats [6]. Because this finding reached significance only in trend analyses and not in pairwise comparisons, it was considered nonconclusive.

The FDA-Other reviews summarized in April 2023 a dose-dependent increase in malignancy cases in participants treated with fezolinetant compared to placebo in the clinical trials. The small number of site-specific cancer cases and poor documentation on pre-existing conditions (eg, such as ultraviolet radiation exposure, underreporting of medical history like past cancer) make it difficult to establish a direct causal relationship between fezolinetant and cancer. According to this document, cancer cases observed could not definitively be attributed to the drug. The FDA concluded that the cancer imbalance may be a chance finding, as no plausible mechanism links fezolinetant to carcinogenesis. Moreover, the short exposure duration makes its contribution to tumor development unlikely [9].

Despite this doubt, the Prescribing Information (PI) of Veozah^®^ does not report neoplasm as a potential unwanted effect and none of the information provided in the FDA review(s) is mentioned in the PI, suggesting the risk is not causally associated.

The EMA Public Assessment Report (EPAR) similarly reported 2 neoplastic cases over 952 participants in the placebo arm, 7 and 9 cases in the fezolinetant 30 and 45 mg treated group, respectively [5]. The discrepancy between the numbers presented in the EMA (16 cases) and FDA (20 cases) documents is already concerning since both agencies were assessing the same studies, but the EMA also mentions that evidence of carcinogenicity was not found in the fezolinetant nonclinical program. Both agencies believed these events were not causally associated with the drug [5, 6].

### Noncausality justification from regulatory documents

Although this risk is not reported in the adverse reaction sections of the US-PI and in the EU-Summary of Product Characteristics (SmPC), the FDA-Clinical Review(s) and the EMA EPAR discussed a potential causal link between fezolinetant exposure and the observed imbalance in the number of cases of neoplasm observed during the SKYLIGHT program [5, 6].

These regulatory documents listed 15 scientific articles [7, 10-23]. According to EMA EPAR, the available literature does not suggest a plausible mechanistic hypothesis by which NK3R antagonism would promote tumor development. Instead, tachykinin agonism may play a role in tumor growth [24].

A careful reading of these articles suggests that 14 out of the 15 do not provide evidence excluding a causal effect (Table 1). Except for Obata et al 2017 [7], discussing the role of NK3R signaling on OSCC, none discuss the link between NK3R antagonism and the reduced risk of neoplasm. The majority (10 out of 13) discuss about neurokinin 1 receptor (NK1R) antagonism [10, 13-18, 20-24], 2 report a NK3R overexpression in OSCC [7, 11] and 1 analyses neurokinin B (NKB), kisspeptin, and their receptors neurokinin 3 (NK3) and kisspeptin in the female genital tract without discussing neoplasm [20]. The choice of reference to support the noncausality is concerning since it seems there is an amalgam between neurokinin 1 and NK3 receptors. Interestingly, an article reviewing patents (2014 to 2020) did not identify any antineoplastic indications for NK3R antagonists [12]. This suggests that this target is not currently associated with any reduced risk of neoplasm, even OSCC, but, as highlighted by the SKYLIGHT and MOONLIGHT programs, fezolinetant, an NK3R antagonist, may be associated with an increased risk [25, 26].

**Table 1:** Analysis of the literature review provided in regulatory documents on biological mechanisms of neoplasm

Year	First author	Title	Summary	Conclusion	Supportive of a causal effect?
2017	Obata	Role of Neurokinin 3 receptor signaling in oral squamous cell carcinoma	The NK3R exhibits varied expression in the central nervous system and human oral squamous cell carcinoma, yet its role in the latter remains unclear. This study examined NK3R expression in surgically resected oral squamous cell carcinoma and its function using a NK3R antagonist in a mouse model of oral squamous cell carcinoma bone invasion. Results showed significant NK3R expression in tumor cells invading the bone matrix, with the NK3R antagonist effectively suppressing osteolytic lesions and tumorigenesis, suggesting NK3R signaling as a potential target for treating oral squamous cell carcinoma with bone destruction.	Data are supportive of NK3R signaling as a potential target for oral squamous cell carcinoma.	No
2014	Covenas	Cancer progression and substance P	The SP/NK1 receptor system drives cancer progression, but NK1R antagonists like aprepitant can counteract this by inhibiting tumor growth and metastasis. Aprepitant, already used clinically for nausea, shows promise for cancer treatment, pending human trials. This approach offers broad potential for improving cancer therapy.	Does not discuss NK3R antagonists - Relates to NK1R.	No
2019	Dikmen	Evaluation of the antileukemic effects of neurokinin-1 receptor antagonists, aprepitant, and L-733,060, in chronic and acute myeloid leukemic cells	NK1R antagonists, including aprepitant and L-733,060, are known for their anxiolytic, antiemetic, anticancer, and anti-inflammatory properties, with aprepitant commonly used to alleviate chemotherapy-induced vomiting and nausea. Both aprepitant and L-733,060 demonstrate strong antiproliferative effects and induce apoptosis in acute and chronic myeloid leukemic cells, suggesting their potential as therapeutic agents for leukemia treatment. These findings highlight the promising role of NK1R antagonists in leukemia therapy and support further exploration of their efficacy in clinical settings.	Does not discuss NK3R antagonists - Relates to NK1R.	No
1995	Hennig	Substance-P receptors in human primary neoplasms: tumoral and vascular localization	The study found SP receptors in specific human neoplasms, primarily in astrocytomas, glioblastomas, medullary thyroid carcinomas, breast carcinomas, and ganglioneuroblastomas, while they were rare or absent in others like nonsmall-cell lung carcinomas, neuroblastomas, and pancreatic adenocarcinomas. These receptors were mainly localized on intra- and peritumoral blood vessels and showed characteristics of the neurokinin-I receptor subtype, suggesting potential clinical implications for diagnosis and treatment, including the use of substance-P antagonists to target tumor vasculature.	Does not discuss NK3R antagonists - Relates to NK1R.	No
2016	Ma	Substance P promotes the progression of endometrial adenocarcinoma	The study aimed to investigate the role of SP and NK1R signaling in endometrial adenocarcinoma progression. It found significantly higher expression levels of SP and NK1R in endometrial adenocarcinoma tissues and cells compared to normal endometrium. SP was found to enhance cell proliferation and invasion of Ishikawa cells, and it induced the expression of matrix metalloproteinase 9 and VEGF-C. These effects were inhibited by NK1R antagonist, suggesting a potential therapeutic strategy for blocking SP-induced promotion of endometrial carcinoma development.	Does not discuss NK3R antagonists - Relates to NK1R.	No
2021	Mehboob	Prognostic significance of substance P/Neurokinin 1 Receptor and its association with hormonal receptors in breast carcinoma	The study investigated the expression of SP and its NK1R in 34 breast carcinoma samples using immunohistochemistry. SP and NK1R were overexpressed in most tumors (68%). The study found a significant association between SP/NK1R expression and tumor progression. The authors suggest that SP/NK1R signaling may contribute to tumor proliferation, angiogenesis, and metastasis, supporting its relevance as a prognostic biomarker and a potential therapeutic target, notably for NK1R antagonists.	Does not discuss NK3R antagonists - Relates to NK1R.	No
2010	Munoz	The NK1 receptor is expressed in human melanoma and is involved in the antitumor action of the NK1 receptor antagonist aprepitant on melanoma cell lines	The study aimed to investigate the presence of NK1R in human malignant melanomas and the potential antitumor action of the NK1R antagonist, aprepitant. Immunoblot analysis and immunohistochemistry confirmed the presence of NK1R in both melanoma samples and cell lines. Additionally, mRNA expression for NK1R was observed in melanoma cell lines, and knockdown experiments indicated their involvement in tumor cell viability. Furthermore, aprepitant treatment resulted in concentration-dependent growth inhibition of melanoma cells, mediated through the NK1R, and induced apoptosis. These findings suggest the NK1R as a promising target and aprepitant as a potential candidate for treating human melanoma.	Does not discuss NK3R antagonists - Relates to NK1R.	No
2018	Nizam	Differential consequences of neurokinin receptor 1 and 2 antagonists in metastatic breast carcinoma cells; Effects independent of Substance P	SP primarily acts through NK1R, followed by NK2R, and their aberrant expression in various carcinomas suggests a role in cancer progression. In this study, highly selective NK1R and NK2R antagonists were examined in metastatic and nonmetastatic breast carcinoma cells, revealing differential responses in cell growth and signaling pathways, indicating potential therapeutic value in targeting these receptors for metastatic breast cancer treatment.	Does not discuss NK3R antagonists - Relates to NK1R.	No
2020	Nizam	NK1R antagonist decreases inflammation and metastasis of breast carcinoma cells metastasized to liver but not to brain; phenotype-dependent therapeutic and toxic consequences	SP acts on NK1R and NK2R, with NK1R inhibitors considered safe and effective for cancer treatment. However, systemic NK1R inhibition may suppress cytotoxic anti-tumoral immune responses. In a syngeneic carcinoma model using metastatic breast cancer cells, highly potent and selective NK1R antagonists showed differential effects on liver metastasis depending on the subset of metastatic cells, suggesting the importance of understanding tumor-microenvironment interactions for effective cancer therapy.	Does not discuss NK3R antagonists - Relates to NK1R.	No
2018	Xu	Substance P attenuates hypoxia/reoxygenation-induced apoptosis via the Akt signaling pathway and the NK1-receptor in H9C2Cells	Myocardial ischemia/reperfusion injury contributes significantly to coronary heart disease-related morbidity and mortality, leading to cardiomyocyte death, compensatory cardiac hypertrophy, dysfunction, and heart failure. While SP is recognized for its cardio-protective effects primarily through coronary vasodilation, its direct impact on cardiomyocytes remains debated. In this study utilizing a hypoxia/reoxygenation cell death model, SP pretreatment was found to inhibit apoptosis in H9C2 cardiomyocytes via the Akt signaling pathway, with involvement of the NK1R, highlighting its potential as a therapeutic target for attenuating I/R-induced myocardial injury.	Does not discuss NK3R antagonists-relates to NK1R.	Yes
2017	Gao	Difference in expression of 2 neurokinin-1 receptors in adenoma and carcinoma from patients that underwent radical surgery for colorectal carcinoma	The study compared expression levels of fl-NK1R and tr-NK1R in colorectal adenoma and carcinoma tissues following surgery. Results showed a significant upregulation of tr-NK1R mRNA in carcinoma tissues compared to adenoma tissues, while fl-NK1R levels remained unchanged. Total NK1R protein levels increased in adenoma and carcinoma tissues, suggesting tr-NK1R's potential role in colorectal adenoma progression and as a diagnostic marker.	Does not discuss NK3R antagonists - Relates to NK1R.	No
2016	Obata	Tachykinin receptor 3 distribution in human oral squamous cell carcinoma	Tac3 and its receptor TacR3, primarily found in the central nervous system and associated with physiological development and human reproductive function, have unknown roles in OSCC. In this study, TacR3 expression was significantly elevated in OSCC tumor cells compared to normal epithelium, particularly at the invasive front into the mandible bone matrix. Conversely, Tac3 was not detected in tumor cells but was present in PGP-9.5-positive sensory nerves in the mandible, suggesting a potential role for peripheral sensory nerve-derived Tac3 in gingival OSCC development.	No causal link that NK3R antagonist reduces the jawbone invasion, there is only a correlation between NK3R expression and lower cancer survival rate.	No
2021	Yoshida	Expression of neurokinin B receptor in the gingival squamous cell carcinoma bone microenvironment	Gingival SCC often infiltrates the maxillary or mandibular bone, with bone destruction being a critical prognostic factor. NK3R, identified as expressed in osteoclasts, has unclear implications for gingival SCC prognosis. In a study of 27 patients, higher NK3R expression in tumor cells correlated with jawbone invasion, while its presence did not significantly relate to tumor size, stage, or differentiation. However, a higher number of NK3R-positive osteoclasts at the tumor bone invasion front significantly correlated with lower disease-specific survival rates, suggesting NK3R as a potential therapeutic target in advanced gingival SCC with bone destruction.	No causal link that NK3R antagonist reduces the jawbone invasion, there is only a correlation between NK3R expression and lower cancer survival rate.	No
2012	Cejudo Roman	Analysis of the expression of neurokinin B, kisspeptin, and their cognate receptors NK3R and KISS1 receptor in the human female genital tract	The study aimed to investigate the presence of NKB/NK3R and kisspeptin/KISS1R mRNA and proteins in the human female genital tract. Samples were collected from reproductive-age and postmenopausal women, and the expression of NKB/NK3R and kisspeptin/KISS1R was assessed using reverse-transcription polymerase chain reaction and immunohistochemistry. Results showed expression of NKB/NK3R and kisspeptin/KISS1R in the uterus, ovary, and oviduct, with coexpression observed in some non-neuronal cell populations, suggesting a potential modulatory role of NKB and kisspeptin in peripheral reproductive tissues.	No mention of tumor, neoplasm or cancer.	No

The EPAR concludes that the preponderance of available literature does not suggest a plausible mechanistic hypothesis for the role of NK3R antagonism in the development of neoplasms [7, 10, 11, 13-20, 22-24].

Abbreviations: fl-NK1R, full-length neurokinin-1 receptors; KISS1R, kisspeptin receptor; NK1, neurokinin 1; NK1R, neurokinin 1 receptor; NK2R, neurokinin 2 receptor; NK3R, neurokinin 3 receptor; NKB, neurokinin B; OSCC, oral squamous cell carcinoma; SCC, squamous cell carcinoma; SP, substance P; Tac3, Tachykinin 3; Tac3R, Tachykinin 3 receptor; tr-NK1R, truncated neurokinin-1 receptors; VEGF-C, vascular endothelial growth factor C.

## Meta-analysis and pooled analysis on the risk of neoplasm with the neurokinin 3 receptor antagonist fezolinetant

A meta-analysis of the SKYLIGHT program, encompassing SKYLIGHT 1, 2, and 4, assessed the neoplasm risk in women with menopause treated with fezolinetant [25]. Findings confirmed a numerical imbalance in the incidence of neoplasms. Particularly, the 52-week SKYLIGHT 4 trial reported neoplasm events in 0.82% and 1.48% of participants receiving fezolinetant 30 and 45 mg, respectively, compared to 0.33% in the placebo group. The baseline incidence in the SKYLIGHT program aligns with the expected annual neoplasm occurrence in the targeted demographic within the United States, supporting the imbalance came from the abnormal incidence in the fezolinetant arm and not from a lower than expected baseline incidence in the placebo arm [25, 27]. Further detailed within the 12-week and extended 52-week phases of the SKYLIGHT 1 and 2 studies, identified neoplasm rates of 1.73% and 1.20% in the fezolinetant 45 mg group, respectively [25]. The meta-analysis revealed a significantly elevated odds ratio (OR) for neoplasm development in participants administered fezolinetant, particularly pronounced at the 45 mg dosage, implying a potential dose-dependent risk increase (ie, OR 4.25, 95% confidence interval (CI): 1.67-10.80) [25]. Approximately half of the reported neoplasm cases were related to skin or mucosal abnormalities, underscoring the necessity for an in-depth investigation to elucidate underlying risk factors and to formulate effective risk minimization strategies. This meta-analysis has been published online on October 18, 2023, ie, after the approval from the EU and US authorities. An additional Kaplan–Meier analysis of the cases reported in the FDA-Clinical Reviews supported the increased risk with a hazard ratio of 4.64 (95%CI, 1.49-14.4; *P* = 0.0081) [26].

Recently, Kagan et al published a pooled analysis of the SKYLIGHT program reporting data on the safety and tolerability of fezolinetant [28]. This analysis included new data on benign and nonbenign neoplasms. One case of nonbenign neoplasm was reported in the placebo group, compared to 6 cases and 13 cases in the fezolinetant 30 and 45 mg group, respectively [28]. The exposure adjusted incidence rate (EAIR) for nonbenign neoplasms was 1.4, 0.7, and 0.2 per 100 subject-year for fezolinetant 45 mg, 30 mg, and placebo, respectively [29]. The relative risk (RR) of these EAIR led to a statistically significant increased risk of neoplasm (RR: 7.83, 95% CI: 1.03-59.66, *P* = 0.0471) [29]. Conversely, the risk for benign neoplasms remains unchanged with no significant difference from placebo.

Development of fezolinetant also includes 2 phase-3 trials in Asia, MOONLIGHT-1 and MOONLIGHT-3, which evaluate its efficacy and safety [30, 31]. MOONLIGHT-1 was a 12-week double-blind study of fezolinetant 30 mg vs placebo followed by 12-week open-label extension [30]. MOONLIGHT-3 was a 52-week open-label study with fezolinetant 30 mg and no comparator arm [31]. Neoplasm data have been published: MOONLIGHT-1 reported 4 cases (2.7%) of “*endometrial hyperplasia/cancer or disordered proliferative endometrium”* in the fezolinetant group compared to 3 cases (2.3%) in the group that initially received placebo but then switched to fezolinetant. No cases of neoplasms occurred in the first 12 weeks, suggesting that all neoplasm cases occurred while on fezolinetant therapy [30]. In MOONLIGHT-3, 5 cases (3.3%) of “*endometrial hyperplasia/cancer or disordered proliferative endometrium”* and 1 case (0.7%) of “*endometrial adenocarcinoma”* were reported [31]. The calculated EAIR was 5.20 per 100 subject-years, exceeding the expected incidence in the general population.

## Potential causality link between the NK3/kisspeptin pathways and their correlation with cell behavior and cancer

### Distribution of neoplastic cases

**Figure 1:**
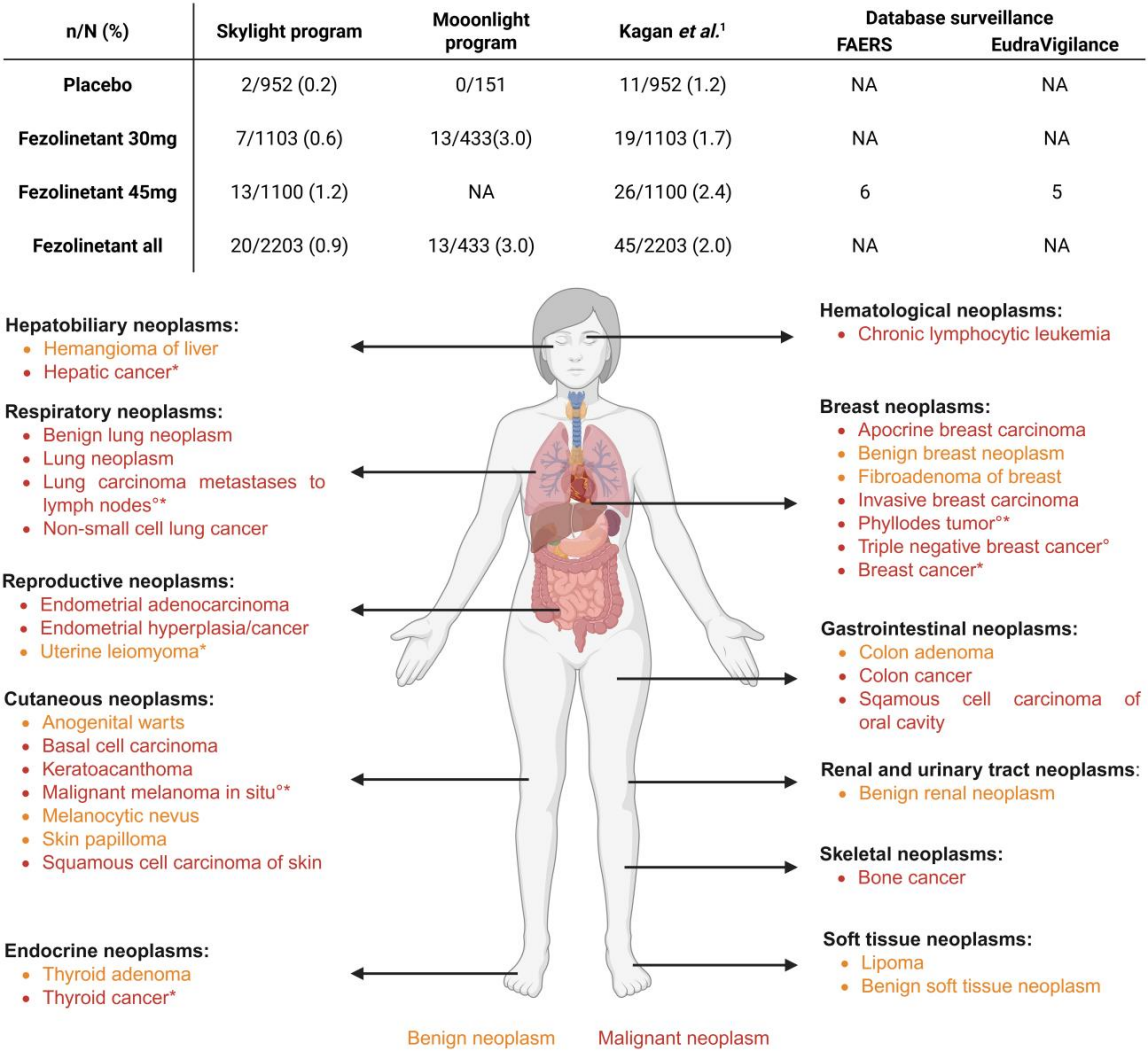
Reported cases of neoplasms with fezolinetant. Data were retrieved from the FDA-Clinical Review (SKYLIGHT program), the MOONLIGHT program, and the pooled analysis by Kagan et al The most frequently *observed neoplasms* are reproductive (*n* = 31), breast cancer (*n* = 10), and skin (*n* = 9). Since the marketing authorization of Veozah^®^, the FDA Adverse Event Reporting System (FAERS) database (data lock point has documented 6 neoplastic cases: phyllodes tumor, thyroid and hepatic cancer, breast cancer, uterine leiomyoma, malignant melanoma, and lung carcinoma with lymph node metastases). In the EudraVigilance database, a case of phyllodes tumor, malignant melanoma, triple negative breast cancer with metastases to the liver, a lung carcinoma metastasis to lymph nodes and a case of (unknown) neoplasm were reported post-authorization in Europe. Red: malignant neoplasms; Orange: benign neoplasms; ^1^: Included benign and nonbenign neoplasms; °: case reported in the EudraVigilance database; *: case reported in the FDA Adverse Event Reporting System (FAERS) database. Data were extracted on July 24, 2025 [6, 28, 30-33]. Abbreviations: *n*, number of neoplasms; *N* safety population for that arm.

A wide range of neoplastic types was reported throughout the clinical development of fezolinetant (Fig. 1), which regulatory authorities interpreted as evidence against a direct causal relationship between fezolinetant exposure and tumorigenesis [5, 6, 28, 30, 31]. However, it may suggest that fezolinetant does not promote oncogenesis through tissue-specific accumulation or direct genotoxic effects, but rather through indirect mechanisms. It may interfere with immunological surveillance or disrupt cellular pathways responsible for suppressing malignant transformation and maintaining tumor cell dormancy.

### Role of NK3R signaling and its functional interaction with kisspeptin

NK3R are peptide receptors belonging to the class A (Rhodopsin) family of G protein-coupled receptors (GPCR) encoded by the TACR3 gene in humans, consisting of an extracellular domain, 7 transmembrane domains, and intracellular cytosolic domains ubiquitously expressed across multiple organs, including the ovaries, uterus, digestive system, and the central nervous system (CNS). NKB binds and activates NK3R, a GPCR, leading to a downstream activation of protein kinase C (PKC)/calcium (Ca^2+^) and protein kinase A (PKA)/cyclic adenosine monophosphate (cAMP) signal-transduction pathways *in vitro* [34]. It plays a critical role in various physiological processes such as reproduction, thermoregulation, vasopressin release, nociception, and neuropsychiatric regulation [34].

NKB, the endogenous ligand of NK3R, regulates the gonadotropin-releasing hormone (GnRH) secretion in the hypothalamic-pituitary-gonadal (HPG) axis in humans via KNDy neurons, which coexpress kisspeptin, NKB, and dynorphin A [34]. NKB binds to NK3R on KNDy neurons, promoting kisspeptin release, activating GnRH neurons and triggering the secretion of the luteinizing hormone (LH) and follicle-stimulating hormone (FSH) [35]. In contrast, dynorphin A acts as a negative regulator by inhibiting KNDy neuron activity, contributing to a feedback loop modulating the frequency of GnRH pulses [36]. Kisspeptin, initially named metastin and discovered in 1996 in Hershey (United States) as a metastasis suppressor in human malignant melanoma, is a crucial neuropeptide that regulates GnRH secretion and plays a key role in reproduction and puberty [37]. Outside the CNS, NK3R roles are less defined, suggesting contributions to intestinal motility, nociception, and pro-inflammatory responses, particularly in the lungs [38-40]. To date, significant gaps remain in understanding the NK3R signaling pathway and its contribution to tumorigenesis.

### Role of kisspeptin pathway in cancer

**Figure 2:**
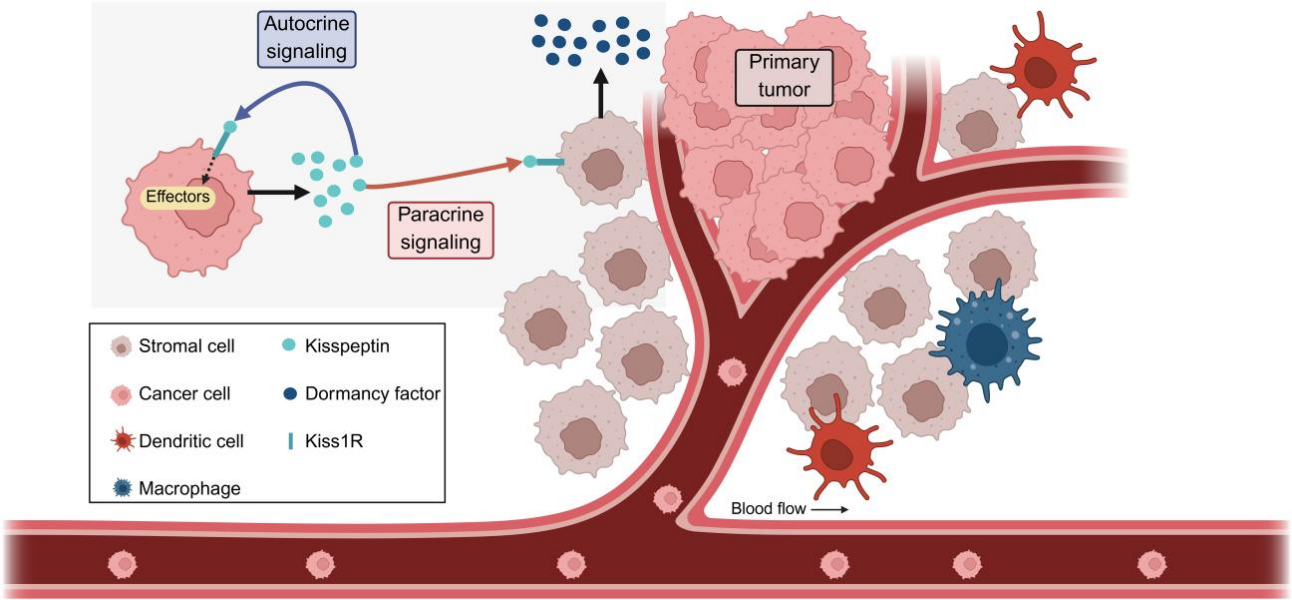
Potential mechanisms of kisspeptin inhibiting metastasis. Kisspeptin has been shown to inhibit metastasis by inducing cancer cell dormancy. Some studies have reported that Kisspeptin receptor (KISS1R) may not be expressed in all cell lines where kisspeptin suppresses metastasis. It suggests that kisspeptin may also act on KISS1R expressed in the microenvironment through a paracrine mechanism. (i) Autocrine signaling: cancer cells secrete kisspeptin, which can then bind to KISS1R on the same cells, activating downstream effectors involved in the regulation of metastasis, proliferation, or tumor progression. (ii) Paracrine signaling: Kisspeptin secreted by tumor cells may bind to KISS1R on neighboring stromal or immune cells. This activation could lead to the secretion of currently unidentified dormancy-inducing factors, which then act on the tumor cells to promote a dormant state [44, 45].

The kisspeptin signaling pathway plays a crucial role in modulating cancer progression by inhibiting tumor cell proliferation, migration, and metastasis. Kisspeptin, encoded by the KISS-1 gene, acts through its receptor to activate the phospholipase C (PLC) pathway, increasing intracellular Ca^2+^ levels and stimulating PKC activation, promoting cellular differentiation, reducing proliferation, and inducing apoptosis [41, 42]. Activation of the kisspeptin/kisspeptin receptor (KISS1R) system exerts multiple effects on cancer cell biology, including suppression of motility, culture scratch repair, proliferation, metastasis, and invasion of human cells *in vitro* [43]. Kisspeptin and its receptor KISS1R have been shown to inhibit multiple steps of the metastasis cascade and to induce cancer cell dormancy by 2 potential mechanisms illustrated in Fig. 2.

It can act autocrinally by binding to KISS1R on cancer cells to activate PLC, generating inositol 1,4,5-trisphosphate (IP3), stimulating the release of intracellular Ca^2+^, reducing cell motility, promoting apoptosis, and stabilizing the cytoskeleton. Kisspeptin may also act in a paracrine manner on stromal and immune cells, maintaining cancer cell dormancy as KISS1R is not always expressed on tumor cells [44, 45].

Kisspeptin inhibits metastasis by blocking focal adhesion kinase and reducing matrix metalloproteinase-9 activity, limiting invasion and extracellular matrix degradation [41, 42, 46]. It also decreases calcineurin activity, a process involved in cellular migration and proliferation [47]. Their antimetastatic activity has been demonstrated across several cancers, including melanoma, thyroid, ovarian, bladder, gastric, pancreas, and lung cancer [48-53].

Clinically, loss or downregulation of the KISS-1 gene correlates with tumor aggressiveness and poor prognosis across multiple cancer types, notably in gastric and ovarian cancer [41, 54]. Overall, enhancing kisspeptin signaling or restoring KISS-1 gene expression holds promise for limiting cancer spread and metastasis. By targeting this pathway, novel strategies can be developed to control cancer progression and improve patient outcomes [41, 54].

### Potential link between NK3 antagonist and NK1R activation

Tachykinins act through 3 GPCRs: NK1R, neurokinin 2 receptor (NK2R), and NK3R, exhibiting a preferential affinity. Cross-reactivity is observed because their shared C-terminal activation motif (-Phe-X-Gly-Leu-Met-NH_2_), whereas the N-terminal region contributes to receptor selectivity. This structural overlap underscores the importance of developing receptor-selective ligands to minimize off-target effects and raises the possibility that selective NK3R antagonism may lead to compensatory activation of the remaining receptors, potentially resulting in unintended pharmacological effects [55].

Because fezolinetant selectively blocks NK3R, unbound NKB may activate the NK1R, which promotes cell proliferation, angiogenesis, and metastasis [21, 23]. This is supported by evidence demonstrating the antineoplastic potential of aprepitant, a selective NK1R antagonist [21, 23]. Physiologically, the NK1R pathway is activated by its preferential ligand, substance *P* (SP). SP belongs to the tachykinin family, which is widely distributed in the body through the nervous and immune systems. It exerts physiological processes and is implicated in various pathological conditions such as pain, inflammation, cancer, or vomiting reflex [56].

Based on data collected from clinical trials and pharmacological hypotheses, namely (i) a potential reduction in kisspeptin levels that could maintain cancer cells' dormancy, (ii) unbound NKB activating the NK1R pathway, and (iii) the widespread distribution of NK3R and KISS1R throughout the body, there appears to be a plausible association between fezolinetant and an increased risk of neoplasms.

### What are the potential mechanisms to support the risk of neoplasm?

Pal et al (2006) show that NKB acts as a reversible inhibitor of endothelial vascular network formation and suppresses angiogenesis. Consequently, disruption of endogenous NKB promotes angiogenic processes [57]. Further evidence by Wang et al (2017) demonstrated the potential anti-angiogenic properties of NKB and its analogues ([MePhe7]NKB, NK3R-A1, and NK3R-A2) exert anti-angiogenic effects through activation of the NK3R in human umbilical vein endothelial cells (HUVEC). Authors demonstrated that NKB treatment significantly decreased vascular density and bed area compared to controls. In a pseudowound assay, NK3R agonists inhibited endothelial cell migration with more than 50% of the wound remaining open compared to the control group where it was nearly completely closed after 24 hours. These findings highlight the role of NKB in modulating angiogenesis. In parallel, the antimigration effect has been demonstrated in the same study using the transwell migration assay [58].

*In vivo,* NK3R agonists reduced tumor volume by half with a decrease in microvessel density on S180 sarcoma cells extracted from mice [58]. Moreover, the NK3R antagonist, [Gly6]NKB[3-10], could antagonize the antiangiogenic effects and the antimigration activity of [MePhe7]NKB, underscoring the important role of NK3R for antiangiogenic and antimigration activity [58].

### What is the potential mechanism that does not support this risk?

One research group suggests that NK3R contributes to OSCC progression, particularly in bone-invasive tumors. Obata et al (2016) demonstrated overexpression of NK3R in OSCC cells invading the mandibular bone matrix and is absent in normal oral epithelium. NKB was undetectable in tumor cells but present in sensory nerves within the mandible, suggesting paracrine signaling between neural elements and carcinoma cells at the bone-tumor interface. This spatial specificity implies that NK3R activation in OSCC is microenvironment-dependent, driven by ligand release from adjacent nerves rather than autocrine production [19]. Moreover, Yoshida et al revealed elevated NK3R expression in gingival squamous cell carcinoma tumors exhibiting cortical and medullary bone invasion, with receptor localization restricted to infiltrative tumor cells at the osteolytic front [11].

The work from Okayama University implicates Sonic Hedgehog (Shh)-mediated autocrine signaling in NK3R-driven osteolysis, where carcinoma-derived Shh activates NK3R to enhance tumor proliferation and bone destruction [11]. Additionally, this research team proposed a potential link between bone resorption and NK3R upregulation, positing that estrogen and other growth factors released during osteoclastic activity may regulate receptor expression via estrogen-related receptor alpha overexpressed in OSCC and known to promote metastasis [19]. However, the link between bone-derived factors, estrogen-related receptor alpha, and NK3R remains unresolved, underscoring the need for further investigation into these microenvironmental crosstalk.

## Clinical implications and recommendations for a safer use of fezolinetant

Given that up to 80% of women with menopause experience VMS and around 25% take treatment, even a modest increase in the absolute risk of adverse events linked to fezolinetant could have a substantial overall clinical impact [59, 60].

As a nonhormonal alternative, millions of women may be exposed to fezolinetant. With an estimated number needed to harm of approximately 80, one additional case of cancer could occur for every 80 women treated. Given the evidence suggesting a potential increase in neoplasm risk, the current regulatory stance, characterized by the absence of any additional postmarketing safety measures, is concerning. Neither the FDA nor the EMA required a PASS, a patient registry, nor specific pharmacovigilance activities focused on malignancies. The FDA-Clinical Review and subsequent documents acknowledge a dose-dependent increase in malignancy rates, yet justify their passive stance based on the heterogeneity of tumor types, short exposure durations, and lack of a clearly identified mechanism. These justifications are speculative and fail to account for (i) the potential indirect oncogenic mechanisms (eg, interference with kisspeptin signaling, NK1R compensation), (ii) the limited statistical power to detect rare or long-latency events in premarketing trials which in this case already detect a signal, and (iii) the high prevalence of hormone-sensitive tissue neoplasms in the reported cases (eg, reproductive tract, breast, and skin). Importantly, the FDA's conclusion that it's a chance finding is not substantiated by long-term safety data. This position neglects the precautionary principle that should prevail in the face of plausible pharmacological mechanisms and current observations from clinical trials. To ensure safer use of fezolinetant and address these uncertainties, risk minimization strategies are listed in Table 2.

**Table 2:** Summary of regulatory action to document and minimize the risk of neoplasm with fezolinetant

Recommended Action	Rationale
Initiate a PASS	To prospectively evaluate the incidence and spectrum of neoplasms over the long term, stratified by dose and patient profile. This is essential given the first-in-class status and potential for class-wide effects, ie, NK3R antagonist.
Establish a Cancer Surveillance Registry	To track neoplasm cases in real-world practice, especially those affecting hormone-sensitive tissues (eg, breast, endometrium, skin), and capture relevant confounders (eg, prior cancer, family history).
Update the SmPC and US PI	Neoplasm risk, though not yet confirmed by regulatory statement, should be transparently reported to prescribers and patients under the *Warnings and Precautions* section to support informed decisions.
Restrict use in high-risk populations	Until more robust data is available, prescribing should be cautious in individuals with personal or familial history of cancer, even if currently there is not sufficient data to claim is the risk is higher in patients with previous neoplastic conditions or family history.
Conduct periodic external safety reviews focused on neoplasm	Annual independent reassessment of global pharmacovigilance data on cancer risk could help identify trends early and ensure regulatory risk minimization measure if needed

Abbreviations: NK3R, neurokinin 3 receptor; PASS, Post-Authorization Safety Study; PI, prescribing information; SmPC, summary of product characteristics.

## Conclusion

Fezolinetant is an effective nonhormonal treatment for moderate to severe VMS but raises concerns regarding a potential increased neoplasm risk, particularly at the 45 mg dose. Pharmacological data discussed in this manuscript suggest that NK3R antagonism could disrupt pathways to tumor suppression, such as kisspeptin signaling, while possibly increasing compensatory activation of oncogenic NK1R pathways. Experimental evidence supports the antiangiogenic and antimigratory roles of NK3R agonism, implying that NK3R blockade may remove physiological restraints on tumor development.

Regulatory authorities have not established a causality between neoplasm and fezolinetant, supporting the absence of any warning in the US PI and the EU-SmPC. No PASS has been requested to specifically monitor the risk. Unresolved uncertainties and mechanistic plausibility, nonclinical studies, long-term safety monitoring, and dedicated real-world evidence studies are urgently warranted to fully characterize the neoplastic risk profile of fezolinetant and ensure appropriate patient protection.

## Data Availability

Data sharing is not applicable to this article as no datasets were generated or analyzed during the current study.
